# The educational use of social networking sites among medical and health sciences students: a cross campus interventional study

**DOI:** 10.1186/s12909-022-03569-3

**Published:** 2022-07-03

**Authors:** Nihar Ranjan Dash, Ahmed Alrazzak Hasswan, Jacqueline Maria Dias, Natasya Abdullah, Mohamed Ahmed Eladl, Khaled Khalaf, Ajmal Farooq, Salman Yousuf Guraya

**Affiliations:** 1 Clinical Sciences Department, College of Medicine, University of Sharjah, P Box - 27272, Sharjah, United Arab Emirates; 2 Department of Nursing, College of Health Sciences, University of Sharjah, Sharjah, United Arab Emirates; 3 Department of Basic Medical Sciences, Faculty of Medicine and Health Sciences, Universiti Sains Islam, Nilai, Malaysia; 4 Department of Basic Medical Sciences, College of Medicine, University of Sharjah, Sharjah, United Arab Emirates; 5 Department of Preventive and Restorative Dentistry, College of Dental Medicine, University of Sharjah, Sharjah, United Arab Emirates; 6 Institute of Dentistry, University of Aberdeen, Aberdeen, UK; 7 Department of Surgery, Ameer-Ud-Din Medical College, Lahore, Pakistan

**Keywords:** Social networking sites, SNSME, Medical education, Curriculum

## Abstract

**Background:**

In recent years, social networking sites (SNSs) have evolved beyond connection and networking to become a powerful instructional tool. There is still a dearth of knowledge on the professional use of SNSs for education particularly among students from diverse backgrounds. This study examined the extent and pattern of SNSs usage for education across six institutions and then conducted an interventional workshop to fortify and regulate the educational use of SNSs.

**Methods:**

This multicenter study was done in two phases. In the first phase, an online cross-sectional survey using a validated inventory was administered to determine the prevalence, extent, and preferences of SNSs usage by undergraduate students in medicine, health sciences and dentistry across five centers. Later, the second phase of the study was undertaken in a 75-min guided live workshop about the appropriate use of SNSs in academia. Additionally, pre- and post-test surveys were conducted to assess the impact and outcome of workshop.

**Results:**

Of the 1722 respondents, 1553 (90%) reported using SNSs with the frequency of once a month to three to five times per day for education and to stay in touch with others. Most students agreed with the benefits of SNSs for education mainly in terms of information gathering, networking and collaboration. Twitter, Instagram, and Pinterest were noted as the most preferred SNSs for education. Nevertheless, 63% perceived that proper instruction was required for the efficient use of SNSs. Following the guided workshop, there was a significant improvement in web technology understanding, digital professionalism, skills and knowledge on the productive use of SNSs. Students rated the efficient for conceptual learning, connection to community practice, e-portfolio, and collaborative learning as the top four major teaching and learning strategies, respectively, in the post-workshop survey.

**Conclusion:**

Our study demonstrates that SNSs can be used as learning tools in medical education. However, SNSs usage should be regulated and guided for a more collegial and coherent learning climate in the digital realm. We urge medical educators to integrate SNSs into their courses for a technologically advanced and impactful curriculum.

**Supplementary Information:**

The online version contains supplementary material available at 10.1186/s12909-022-03569-3.

## Background

Social networking sites (SNSs) are online platforms that people use to establish social relationships and build networks with other people who share their personal or professional interests [1]. These sites are developed and secured by Web 2.0 applications which pertain to diverse web-enabled applications created on an open source platform and run by user-generated and user-manipulated content [2]. The most frequently used Web 2.0 applications include wikis (Wikipedia), podcasts (YouTube), blogs (BlogSpot), and SNSs including Twitter, Instagram, Pinterest, TikTok, and Wechat [3, 4]. SNS applications are primarily used to foster friendships, stay in touch, share and exchange information, upload photos, videos, and news feeds [5].

Recently, SNSs have been incorporated into medical education to learn, interact, discuss, collaborate, recruitment and develop professional skills [6]. According to literature, in medical education approximately 75% of learners use some form of SNSs, of which only 20% use SNSs for academic and educational purposes [7]. Faculty use SNSs to post opinions, views, videos, chat, participate in surveys, and even manage some parts of their courses. From an educational standpoint, SNSs are frequently used as novel tools for teaching and learning and for enhancing educational interactions among peers, students, and faculty. The literature points out that Twitter, Facebook and WhatsApp have been instrumental in guiding student assignments and projects, enhancing students’ learning engagements, creating a positive learning climate for education, particularly learning outside the classroom [8, 9].

The recent COVID-19 pandemic has changed the education strategies dramatically across the world [10]. The distinctive rise of e-learning being undertaken remotely has revolutionized the use of digital platforms and SNSs. The successful integration of online learning using digital platforms and SNSs will continue to persist post-pandemic. However, an obvious gap in technology use between the faculty and students, where the z-generation students are quicker to adopt SNSs habitually than the faculty, can create a lag and imbalance between learning and teaching [11]. This digital divide sparks several questions, including whether we should limit SNSs or to follow the students’ preferred learning style as they are tech-savvy and feel more comfortable while learning in a cyber space.

While the frequency, pattern and purpose of SNSs usage have been somewhat deciphered [12], there is still a lack of information about the ethical and efficient use of SNSs by students for academic purposes. Despite the fact that medical educators have provided tips on how to use SNSs, particularly Twitter and Facebook, as learning tools in medical education [13], as well as interventional actions to improve medical students' use of SNSs [14, 15], users continue to struggle with issues of confidentiality, privacy, and e-professionalism in the ever-changing social media environment.

This multi-center study was designed to determine the extent and pattern of SNSs use in education, as well as the impact of an interactive intervention on medical, health sciences, dental, and pharmacy students from the United Arab Emirates (UAE), Malaysia, and Pakistan In addition, the participants were given the opportunity to learn about the available learning tools and features of SNSs, as well as the concept of e-professionalism. The findings of this study have the potential to increase awareness among undergraduate medical students and medical educators regarding their choices of SNSs and electronic professional identities through e-professionalism in response to the ever-changing landscape of social media. 

## Methods

This study was carried out in two phases. In the first phase, a cross-sectional study was conducted to determine the extent, nature, and purpose of SNSs by undergraduate students from a range of health professions. In the second phase, we conducted an interventional workshop which was based on the data and the key findings from the first phase of the study together with a pre-post survey to determine the impact and outcome of the workshop. A convenient sampling method was employed for the recruitment of the undergraduate students across all years from five different centers namely the College of Medicine (CoM) University of Sharjah (UoS) UAE, College of Health Sciences (CHS) UoS, College of Dental Medicine (CDM) UoS, Faculty of Medicine and Health Sciences, Universiti Sains Islam Malaysia (USIM) and Ameer-ud-Din Medical College (AMC) Pakistan. These institutions were selected due to their significant commonalities in the curriculum contents including the courses, teaching and assessment modalities.

### Study settings

The curriculum of the CoM at UoS in the UAE spans over six years and adopts a student-centred problem-based learning strategies. The MBBS curriculum is further divided into three phases: phase I—foundation year, phase II—pre-clerkship phase (years 1, 2 and 3) and phase III—clerkship phase (years 4 and 5). The CHS at the UoS has seven departments: medical laboratory sciences, medical diagnostic imaging, nursing, health services administration, physiotherapy, environmental health sciences, nutrition and diabetes. All seven programs utilize a classical 4-year outcome-based competency curriculum. The CDM at the UoS provides the Bachelor of Dental Surgery (BDS) program in an integrated, theme-based 6-year curriculum. It comprises of three phases: phase I- foundation sciences, phase II- integrated dental sciences and phase III- dental clerkship. The Faculty of Medicine and Health Sciences at USIM Malaysia offers a 6-year medical curriculum that includes three years of pre-clinical training and another three years of clinical posting. The program adopts a discipline-based curriculum that incorporates both directed and student-centered learning. The AMC in Pakistan offers a five-year undergraduate medical program using the classical competency-based, integrated curriculum. Besides the varied geographical locations among all participating institutes, the undergraduate students in these institutes had some kind of social presence and used a wide variety of SNSs to post opinions, share surveys, share videos, post articles and course related material beside networking, entertainment and socialization. However, there is no structured course or teaching pedagogy in any of the participating institutions about the educational use of SNSs for medical and health sciences students.

### Measurement of SNSs usage

In the first phase of the study, the research collaborators from each of the five institutes invited their students to participate in this study through emails. The study instrument was an online questionnaire that was sent via SurveyMonkey® platform. We adopted a previously tested and published English-language 20-statement social networking sites for medical education (SNSME) inventory[12]. The SNSME inventory captures the usage, extent and preferences of students for SNSs. The first six statements of SNSME gathers information about the frequency of the usage of SNSs from five options of never, once a month, once a week, once a day, and 3–5 times per day (Additional file 1: Appendix I). The next 14 statements of the questionnaire capture the responses of the participants about the mechanisms for the usage of SNSs for education on a 5-point Likert scale (e.g., strongly agree, agree, neutral, disagree, and strongly disagree). Data and key finding from this phase was used to develop the intervention workshop for the 2nd phase of the study.

### Intervention workshop development

For the 2^nd^ phase of the study, a 75 -minute guided live workshop was developed and structured around the educational use of the three most common SNSs indicated by the study cohort. The workshop's main goals were to discuss the available features, benefits, limitations, and challenges of using Twitter, Instagram, and Pinterest in medical education, as well as the attributes of e-professionalism and the mechanisms for protecting privacy and confidentiality. We invited the same cohort of students to register for an online guided workshop on the academic usage of SNSs in the medical field. The interested students were required to register through an online Google form. By analyzing the data from the SNSME survey, we came across a host of information about the most popular SNSs and a range of strategies that were adopted by students for the educational use of those SNSs. All registered students were invited to watch a 15-min pre-workshop presentation at their own time and pace though a shared link. The recorded lecture introduced students to Web 2.0 technology and the use of SNSs in medical education.

The workshop was designed with the agenda “academic use of Twitter, Instagram and Pinterest in undergraduate medical education” which was carried out live via MS Teams. During the interventional workshop, after a brief introduction, a 45–minute interactive presentation was delivered. This presentation vividly demonstrated the learning and collaborative strategies available on the three most popular SNSs opted by the students during the first phase. A brief account about e-professionalism and its advantages and disadvantages was also touch-based in this interactive presentation. This was followed by an open questions and answers session of 15 minutes. All researchers attended this workshop as facilitators and participated in groups discussions and in wrap up session. The attending students contributed by raising hands, writing in chat room and as well as by directly speaking to the presenters. During the workshop, participants completed a self-administered pre- and post-intervention questionnaire to assess their perspectives, insights, and the impact of the intervention. The questionnaire had five statements to respond on a Yes or No scale, two multiple-choice questions and one open ended question (Additional file 1: Appendix II).

### Data collection and analysis

The data was entered and analyzed using the Statistical Package for Social Sciences v.23 (SPSS). The quantitative descriptive analysis was done by frequency distributions which was illustrated in graphical and pictorial presentations in clustered bar charts. As all statements were organized in ordinal scale, inferential statistics were performed by non-parametric tests. As a pre-requisite to using other non-parametric tests (Mann–Whitney U and Kruskal Wallis tests), the normality of data was cross-verified by a one-sample Kolmogorov- Smirnov test. If a variable had a significant z value (< 0.05), this would allow us to reject the null hypothesis “data is normally distributed”. Therefore, non-parametric tests would be considered appropriate for the comparison of responses from genders, year of schooling and age groups. The Mann–Whitney U test was used to compare the differences in responses between genders and the Kruskal Wallis test compared the variations between more than two independent groups e.g., year of schooling and age groups. For the pre-post statistical analysis of the guided workshop, a paired t was used. A *p-*value of less than 0.05 was considered significant.

## Results

### Respondent’s background

We received 1722 complete responses out of 1986 invitees (response rate of 86%): 1277 (74%) female students and 445 (26%) male students. The majority of the respondents (843; 48.9%) were between the ages of 18 and 20, 463 (26.9%) were between the ages of 21 and 23, 305 (17.7%) were between the ages of 24 and 27, and 113 (6.6%) students were over the age of 27. There were 515 (29.9%) students from CoM-UoS, 399 (23.2%) from CHS-UoS, 265 (15.4%) from CDM-UoS, 237 (13.7%) from AMC, and 306 (17.8%) students from USIM.Further, the distribution of the students across different years in the colleges are shown in Table 1.

**Table 1:** The breakdown of responses by students across the years of study in the participating institutions

Institution	Total respondent	FY	Y1	Y2	Y3	Y4	Y5	Y6
CoM-UoS	515 (29.9%)	263	73	62	46	24	47	-
CHS-UoS	399 (23.2%)	-	65	92	148	94	-	-
CDM-UoS	265 (15.4%)	142	25	20	22	24	32	-
AMC	237 (13.8%)	-	46	94	24	32	41	-
USIM	306 (17.8%)	-	74	92	62	56	16	06
	1722 (100%)	405 (23.5%)	283 (16.4%)	360 (20.9%)	302 (17.5%)	230 (13.3%)	136 (7.9%)	06 (0.3%)

### Usage, extent and preferences of students for SNSs

Out of the total 1722 respondents, 1553 (90%) used the SNSs for educational purposes with the frequency of usage ranging from once a month to 3–5 times per day (Table 2 panel A). In comparison to other centers, students from CoM-UoS had the highest percentage of active users of SNSs for education (29% of total users). Only a small proportion of respondents (9.8%) had never used SNSs for educational purposes. The majority of respondents (1475/1772 or 86%) agreed or strongly agreed that social networking sites were beneficial for educational reasons as shown in statement 18 (*S18. I have found social networking sites useful for educational purposes*) of Table 2 panel B. However, a small percentage of the respondents (2.5%) disagreed with the statement. Additionally, 1093/1722 (63%) respondents agreed (e.g., strongly agree and agree) that proper counselling was necessary for efficient use of SNSs for education as shown in statement 19 (*S19. Medical students need supervision and guidance for the appropriate use of social networking sites for educational purposes*) (Table 2 panel C). We discovered that the most frequently used SNSs for medical education were Twitter, Instagram, and Pinterest, as indicated from 379 (22 percent), 327 (19 percent), and 310 (18 percent) respondents of the cohort, respectively (Table 2, panel D).

**Table 2:** The extent and pattern of social networking sites usages by the students from all participating institutions

**Responses**	**CoM-UoS**	**CHS-UoS**	**CDM-UoS**	**AMC**	**USIM**	**Total**
**Panel A:** The responses of students to “*S3: how often do you use social networking sites (i.e., Facebook, YouTube, Instagram, Twitter, LinkedIn, and Flickr to share education-related information?*” (*n* = 1722)						
Never	61 (4%)	36 (2%)	31 (2%)	24 (1%)	16 (1%)	168 (10%)
Once a month	41 (2%)	59 (3%)	30 (2%)	22 (1%)	48 (3%)	200 (12%)
Once a week	109 (6%)	75 (4%)	47 (3%)	48 (3%)	68 (4%)	347 (20%)
Once a day	119 (7%)	102 (6%)	78 (5%)	65 (4%)	86 (5%)	450 (26%)
3–5 times a day	184 (11%)	127 (7%)	79 (5%)	78 (5%)	88 (5%)	556 (32%)
**Panel B:** The responses of students to “*S18: I have found social networking sites useful for educational purposes”*(*n* = 1722)						
Strongly agree	219 (3%)	160 (9%)	126 (7%)	115 (7%)	171 (10%)	791 (46%)
Agree	215 (12%)	171 (10%)	90 (5%)	87 (5%)	121 (7%)	684 (40%)
Neutral	60 (3%)	54 (3%)	42 (2%)	27 (2%)	9 (1%)	192 (11%)
Disagree	6 (0.3%)	6 (0.3%)	3 (0.2%)	6 (0.3%)	2 (0.1%)	23 (1%)
Strongly disagree	15 (0.9%)	5 (0.3%)	4 (0.2%)	2 (0.1%)	3 (0.2%)	25 (1%)
**Panel C:** The responses of students to “*S19: medical students need supervision and guidance for the appropriate use of social networking sites for educational purposes”* (*n* = 1722)						
Strongly agree	142 (8%)	120 (7%)	61 (4%)	67 (4%)	135 (8%)	525 (30%)
Agree	120 (7%)	157 (9%)	77 (4%)	107 (6%)	107 (6%)	568 (33%)
Neutral	142 (8%)	80 (5%)	79 (5%)	39 (2%)	52 (3%)	392 (23%)
Disagree	79 (5%)	27 (2%)	30 (2%)	19 (2%)	8 (0.5%)	163 (10%)
Strongly disagree	32 (2%)	12 (0.7%)	15 (1%)	5 (0.3%)	4 (0.2%)	68 (4%)
**Panel D:** The most used social networking sites for education in this study (*n* = 1722)						
Twitter	99 (6%)	80 (5%)	85 (5%)	67 (4%)	48 (3%)	379 (22%)
Instagram	65 (4%)	56 (3%)	55 (3%)	69 (4%)	82 (5%)	327 (19%)
Pinterest	73 (4%)	60 (3%)	71 (4%)	58 (3%)	48 (3%)	310 (18%)
YouTube	69 (4%)	57 (3%)	67 (4%)	55 (3%)	45 (3%)	293 (17%)
LinkedIn	57 (3%)	47 (3%)	55 (3%)	45 (3%)	37 (2%)	241 (14%)
Others	41 (2%)	33 (2%)	39 (2%)	32 (2%)	27 (2%)	172 (10%)

**Fig. 1:**
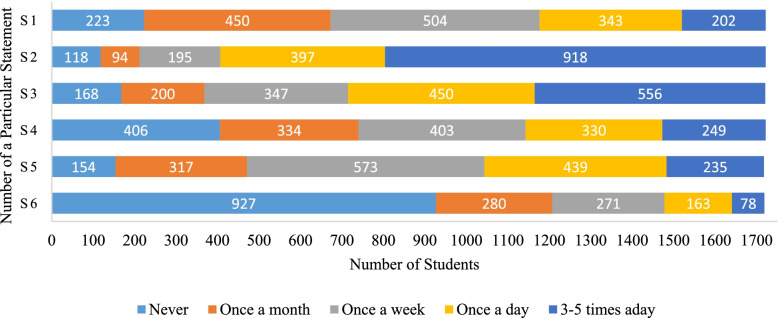
The observed frequencies of responses to statements about the students’ extent of the usage of social networking sites for education (*N* = 1722)

Figure 1 shows the clustered bar chart of the observed frequencies of responses through categorical variables (1^st^ category = never used, 2^nd^ category = once a month, 3^rd^ category = once a week, 4^th^ category = once a day, and 5^th^ category = 3–5 times a day). For the first statement ‘*S1*. *How often do you use e-mail for sharing information for educational purpose?*, we observed that most students (504; 29%) used email once a week for sharing educational material. For second statement ‘*S2.* H*ow often do you use social networking sites to keep in touch with peers and tutors?’* most (918; 53%) students used SNSs to remain in touch with their peers and tutors 3 to 5 times a day. Interestingly, most students (927; 54%) did not contribute to blogs writing as shown by their responses to ‘*S6. How often do you contribute to blogs or Wikis to share information, or for dissemination of knowledge?’*. Overall, students’ response to SNSs usage for education remained mixed.

**Fig. 2:**
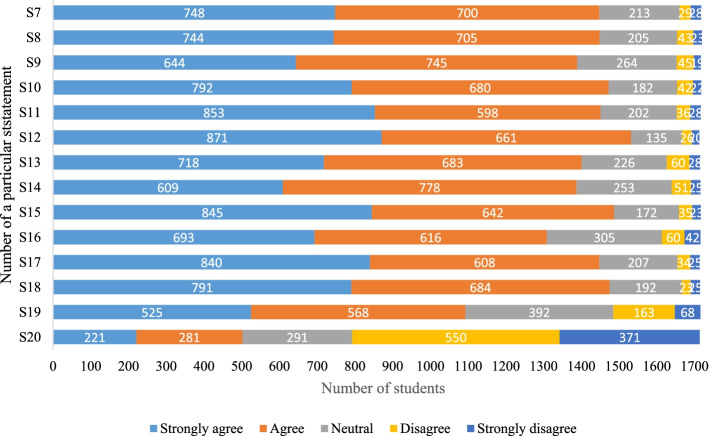
The observed frequencies of responses to statements about the students’ perceptions of the usage of social networking sites for education (*N* = 1722)

Figure 2 displays the bar chart of the observed frequencies of responses to statements about the students’ usage of the SNSs for education using the same categorical variables. The highest response was recorded for statement ‘*S12. Social networking sites help me to access educational resources*’, where most (871; 51%) students strongly agreed that SNSs was an important platform for sharing educational material. On the other hand, for the 20^th^ statement ‘*S20. I believe that social networking sites are inappropriate for sharing classroom materials, information, and discussing education related topics’*, majority of the respondents either disagreed (550; 32%) or strongly disagreed (371;21%). Similarly, the responses to other statements are outlined in Fig. 2.

Table 3 compares the mean ranks of all responses by students across their years of schooling. The results showed that the students’ responses were significantly different for all statements. The responses of foundation year students significantly dominated those from other years as evident by their higher mean ranks e.g., the highest mean rank of 1098 for statement 1. Likewise, the differences in responses to other statements are highlighted in italics. Table 4 compares all statements based on mean ranks across different age groups of students and the results showed that the students’ responses were significantly different from each other for all statements. Senior students with age groups above 27 years scored highest mean rank of 1167 for statement 1. The variations of the other statements are highlighted and italics. Table 5 compares all statements based on mean ranks across different colleges of students. It is noteworthy that senior students from CHS-UoS scored highest mean rank of 971 for statement 1.

**Table 3:** A comparison of students’ responses about the usage of social networking sites for education across years of schooling using the Kruskal Wallis test (*N* = 1722)

Statement	Mean rank							Chi-square	*p*-value
	**FY**	**Y1**	**Y2**	**Y3**	**Y4**	**Y5**	**Y6**		
S1	***1098***	809	833	719	835	717	733	141.66	0.00*
S2	887	859	***905***	897	800	789	775	13.72	0.03**
S3	***927***	898	855	836	818	768	688	23.68	0.00*
S4	***923***	822	873	876	800	771	829	15.88	0.01**
S5	890	820	881	865	832	774	881	8.43	0.21
S6	***972***	821	871	827	818	758	691	44.12	0.00*
S7	834	846	886	813	909	932	844	10.84	0.09
S8	855	820	886	820	889	***971***	820	13.16	0.04**
S9	803	839	913	817	902	916	*934*	18.83	0.00*
S10	835	879	878	820	898	881	822	6.73	0.35
S11	827	845	878	844	893	931	827	7.28	0.30
S12	849	827	885	816	881	942	832	10.05	0.12
S13	853	853	857	787	895	***995***	882	17.90	0.01**
S14	849	859	862	815	872	964	859	8.54	0.20
S15	839	833	883	802	915	***921***	920	13.74	0.03**
S16	***938***	850	854	790	834	817	873	19.69	0.00*
S17	***919***	829	866	791	856	879	800	16.33	0.01**
S18	864	839	885	801	870	920	851	8.69	0.19
S19	***937***	815	890	766	807	882	913	29.72	0.00*
S20	883	***885***	882	852	748	834	828	14.06	0.03**

Here grouping variable is age group, * and ** represents the level of significance at 1% and 5%, respectively

*FY* Foundation year, *Y* Year

S1. How often do you use e-mail for sharing information for educational purpose?

S2. How often do you use social networking sites (e.g., Facebook, Youtube, Twitter, Linkedin, Wechat and Flickr) to keep in touch with peers and tutors?

S3. How often do you use social networking sites (i.e., Facebook, Youtube, Twitter, Linkedin, and Flickr) to share education-related information?

S4. How often do you use social networking sites (i.e., Facebook, Youtube, Twitter, Linkedin, and Flickr) for sharing research, innovations in medicine, and updates in medical field?

S5. How often do you read blogs or Wikis for education related information?

S6. How often do you contribute to blogs or Wikis to share information, or for dissemination of knowledge?

S7. Social networking sites help me in collation of educational materials

S8. Social networking sites are helpful in collaborative and peer-to-peer learning

S9. Social networking sites are useful in developing reading and writing web skills

S10. Social networking sites provide opportunity of virtual meeting with other students and tutors

S11. Social networking sites help me to communicate with peers about class projects

S12. Social networking sites help me to access educational resources

S13. Social networking sites help me to retrieve educational references for research

S14. Social networking sites facilitate my professional development of learning skills in technology

S15. Social networking sites are useful in communicating with classmates about course-related topics

S16. I have found social networking sites useful during the pre-exam period when I get an instant answer/explanation from my peer, instead of going through the books

S17. I have found social networking sites useful for sharing notes and lectures

S18. I have found social networking sites useful for educational purposes

S19. Medical students need supervision and guidance for the appropriate use of social networking sites for educational purposes

S20. I believe that social networking sites are inappropriate for sharing classroom materials, information, and discussing healthcare related topics

**Table 4:** A comparison of the students’ opinions about the usage of social networking sites for education across age groups using the Kruskal Wallis test (*N* = 1722)

Statement	Mean rank				Chi-square	*p*-value
	**18–20 years**	**21–23 years**	**24–27 years**	**Above 27 years**		
S1	924	751	723	*1167*	60.85	0.00*
S2	867	846	832	875	1.16	0.76
S3	889	815	765	***896***	12.67	0.01**
S4	862	861	809	860	1.07	0.79
S5	848	864	922	759	2.95	0.40
S6	885	821	753	***980***	13.92	0.00*
S7	844	877	863	964	2.78	0.43
S8	849	881	800	925	3.73	0.29
S9	845	876	863	857	1.73	0.63
S10	858	859	827	857	0.43	0.94
S11	842	885	851	809	3.50	0.32
S12	854	850	865	951	0.86	0.84
S13	854	845	922	893	2.46	0.48
S14	854	854	887	853	0.47	0.93
S15	848	861	901	954	2.14	0.54
S16	870	839	780	939	4.83	0.19
S17	873	835	761	920	7.17	0.07
S18	863	838	855	925	1.51	0.68
S19	***884***	810	827	795	9.55	0.02**
S20	873	832	826	630	6.52	0.09

Here grouping variable is age group, * and ** represent the level of significance at 1% and 5%, respectively

**Table 5:** The results of the Kruskal Wallis test showing the comparison of the students’ perceptions about the usage of social networking sites for education across the participating institutions (*N* = 1722)

Statement	Mean rank					Chi-square	*p*-value
	CoM-UoS	CHS-UoS	CDM-UoS	AMC	USIM		
S1	911	***971***	956	599	499	263.87	0.00*
S2	850	795	800	740	*903*	19.85	0.00*
S3	866	810	810	799	815	5.59	0.23
S4	841	787	838	730	***901***	17.42	0.00*
S5	837	778	786	713	***961***	41.42	0.00*
S6	822	***962***	862	688	701	80.05	0.00*
S7	841	***903***	811	853	704	36.99	0.00*
S8	810	891	849	***926***	713	37.79	0.00*
S9	815	869	775	***952***	777	22.85	0.00*
S10	810	***919***	813	904	713	43.99	0.00*
S11	790	896	824	***902***	766	24.27	0.00*
S12	824	***904***	852	810	705	38.77	0.00*
S13	810	***888***	880	867	706	36.30	0.00*
S14	819	874	837	***880***	740	18.65	0.00*
S15	829	882	784	867	768	15.69	0.00*
S16	821	877	824	804	780	8.90	0.06
S17	838	***887***	841	829	705	31.93	0.00*
S18	840	883	828	***841***	716	27.05	0.00*
S19	840	883	828	841	716	81.04	0.00*
S20	***881***	702	851	826	861	38.77	0.00*

Here grouping variable is college group, * and ** represent the level of significance at 1% and 5%, respectively

### Pre- post intervention surveys

A total of 143 students attended the guided workshop, however, we retrieved 89 complete responses to the pre-post surveys. There were comparable representations of students from all colleges of UoS, USIM and AMC. The results of the paired t test showed a significance improvement in the students’ understanding and knowledge about the educational use of SNSs by the guided workshop compared to their pre-workshop status (Table 6). Specifically, students’ understanding about Web 2.0 technology and its applications in the digital age improved significantly to 45% compared to 23% in pre-workshop survey (*p* < 0.000). Second, their knowledge about digital professionalism improved from 43 to 83% by the intervention (*p* < 0.000). Finally, students’ skills and knowledge about the productive use of SNSs significantly increased to 91% after the workshop (*p* < 0.00).

**Table 6:** A comparison of the *s*tudents’ pre- post understanding and knowledge about the educational use of social networking sites in this study (*N* = 89)

Statement	Mean pre	Mean post	Mean Diff (post–pre)	t stat	*p*-value
Do you have prior knowledge of Web 2.0 technology and its applications in the digital age?	0.23	0.45	0.22	4.68	0.000
Do you know what “digital professionalism” is?	0.43	0.83	0.39	7.35	0.000
Has this workshop added to your knowledge, and understanding skills in using social networking sites for education?	0.09	0.91	0.83	19.69	0.000

Table 7 shows the rankings of the learning strategies in SNSs by students in order of their preferences. The data showed a significant improvement in the students’ knowledge and understandings about a range of learning modalities on SNSs when compared to their pre-workshop levels (*p*-value 0.007). We observed that students rated *efficient for conceptual learning, connection with community practice, e-portfolio, and collaborative learning* as top-four significant teaching and learning strategies, respectively, in post-workshop. Interestingly, the students’ cohort attending the workshop once again favored Twitter (38/89, 43%), Instagram (22/89, 24%), and Pinterest (20/89, 22%) as the three most popular SNSs being used for their teaching and learning, similar to the initial survey.

**Table 7:** Preferences of students about the usage of social networking sites in medical education

Teaching and learning strategies	Post	Pre	Difference (post–pre)
1. Class participation	58	63	5
2. Connection with experts in your area of study	68	73	5
3. Collaborative learning	55	64	9
4. Up-to-date information	69	69	0
5. Interaction among classmates/peers	55	58	3
6. Efficient for conceptual learning	26	40	14
7. Connection with community practice	41	54	13
8. Feedbacks	43	46	3
9. E-portfolio (Self-documentation of personal work and achievement)	28	38	10
10. Publish ideas and opinions in real-time format	33	40	7
Mean	47.9	54.1	6.2
Paired t-test	t stat. = 3.52		*p*-value = 0.007

## Discussion

Our cross-campus study draws on the use of SNSs which can be transformed by faculty and students from medical and health sciences into an authentic digital footprints where they can work collaboratively within the medical community. Overall, approximately one third of the students’ cohort actively used SNSs for education, while almost one half of the cohort found SNSs as an effective and useful medium for education. The staggering upsurge of the adaptation of global digital applications is clearly fueling the use of SNSs as approximately 45% of the world population is using some kind of social media every day [16].Twitter, Instagram, and Pinterest were the three most popular SNSs choices by our study cohort for their learning activities. Likewise, the study inferred that the students most commonly used SNSs for *conceptual learning, connection with community practice, e-portfolio, and collaborative learning*. Surprisingly, publishing ideas and opinions in real-time was the least preferred learning modality among the study cohort. This could be attributed to the poor writing and publishing skills of students who need further training on critical appraisals and micro-reflections.

In this study, Twitter was found to be the most popular SNS for medical education. By following hashtags, tweeting, and retweeting, students used Twitter to communicate with their peers, tutors, and faculty. Twitter usage promoted a variety of informal learning activities, such as self-directed, independent, and collaborative assignment work [17]. It aided in the formation of e-learning communities in a cyberspace interprofessional milieu, fostering flexible and collegial learning outside of regular work hours, particularly among medical students [18]. Junco et al., investigated the impact of Twitter on college students in 125 pre-health majors and concluded that Twitter had a positive impact on both students’ engagement and assessment grades [19]. On the other hand, a study by Scot et al., found a decline tendency in academic use of Twitter over time, notably in anatomy education [20]. Several possible explanations for this decline have been proposed, including social media fatigue, changing the nature and content of social media platforms, becoming bored and frustrated with a particular platform over time, and the young generation's constant desire to switch to a newer and trending platform, such as moving away from Facebook and Twitter to Instagram and TikTok [21].

The second most popular SNS in our study was Instagram, a smartphone- and a tablet-based program with an image-sharing service, which asynchronously publishes images using a plethora of digital filters [22]. Because of its video and photo upload and sharing capabilities, it is becoming increasingly popular in human anatomy, radiology, and dental education [23, 24].

The majority of Instagram users are young students aged 18 to 29 where they frequently upload informal peer-to-peer study-related material. Although Instagram has its own terms of service, which prohibit the publication of unlawful and confidential content, it still lacks quality control, confidentiality, and ethical and legal regulations for posting sensitive or personal information. Educators have an opportunity and responsibility to guide and engage the young minds in professional, and quality-assured informative in SNSs [20].

Pinterest, the third most popular SNS in our study, is an online service for creating and sharing images with an opportunity to create instructional resources [25]. A classic example of an image-sharing application in Pinterest is CTisus.com, a radiology-teaching website that enables users to browse a host of images of a specific illness with insightful notes and guidance. A great majority of students uses Pinterest to pin (add images), re-pin, comment, describe, and download images and flow chart for the academic activities.

In our study, CoM-UoS had the most active users of SNSs for education (453/514; 88%) when compared to other colleges. This could be due to the  fact that CoM-UoS students had the highest representation, as well as due to their constantly evolving affinity for SNSs. A study in 2014 at the CoM-UoS on the use of a Facebook page in anatomy teaching found a similar effect, with the majority of students embracing and finding it utility for learning [26]. This was followed by another study in 2016 where the authors reported YouTube and Facebook were the top ranked SNSs used by the students in CoM-UoS [27]. Senior students from CHS-UoS received the highest mean rank for their degree of online application connectivity. Likewise, CoM-UoS and USIM students showed the highest agreement with the statement ‘*I have found social networking sites useful for educational purposes*’. Another interesting observation from our research was that senior students above the age of 27 had higher mean ranks than their peers. This could be due to the nature of education, particularly clinical training and increased exposure to medical apps in patient care, thus more empowered to use SNSs professionally based on experiences [28], despite the fact that the process is unsupervised and unstructured.

From our study cohort, responding to the statement, ‘*medical students need supervision and guidance for the appropriate use of social networking sites for educational purposes*’, 63% students agreed for the need of professional training for the educational use of SNSs. There is no disagreement with this finding, although some medical schools offer a structured course or module on the educational use of SNSs [14, 15], the usage of SNSs in education is still inconsistent and fragmented. Furthermore, there have been multiple reports of medical students acting in an unprofessional or questionable manner, breeching privacy, compromising confidentiality, and blurring personal and professional lines, all of which have resulted in uncertain legal ramifications [29, 30]. The live workshop session was held to support students and raise awareness about the use of SNSs in medical education, notably Twitter, Instagram, and Pinterest. Additionally, the educational intervention highlighted the emerging concept of e-professionalism, “attitudes and behaviors (some of which may occur in private settings) reflecting traditional professionalism paradigms that are manifested through digital media” [31]. The interventional workshop, according to the vast majority of students, improved their knowledge of social networking sites for medical education as well as Web 2.0 technology and its applications in the digital sphere. We believe, for a successful use of SNSs in medical education, a thorough review of all SNSs and professional development programs for faculty, healthcare practitioners, and students is required. Finally, all stakeholders should have access to institutional regulations for implementing, maintaining, and monitoring a safe and legal digital policy.

### Study limitations

This study has few potential limitations. First, there was a small sample of students who attended the online session. Despite the small sample size, the engagement and response rates were satisfactory. Second, the limited access to various SNSs, as determined by their local laws and regulations, could have influenced the study findings. Third, a selection bias of the attitudes and practices of the respondents who used SNSs were different from non-respondents who potentially did not use SNSs. Despite these limitations, we believe that this study accomplished its objectives of measuring SNSs usage among medical and health sciences students and in guiding them for their better educational application.  

## Conclusions

In conclusion, among undergraduate students in medicine and health professions, Twitter, Instagram, and Pinterest remained the top popular SNSs for academic usages. Their applications are currently highly inconsistent and personalized. In comparison to the traditional and orthodox teaching and learning pedagogies, however, the future of SNSs in academia appears promising and powerful. Through involvement, collaboration, peer supported learning, and feedback, SNSs might potentially improve students' learning experiences. As the pedagogical benefits of SNSs are currently only partially realized, there is a room for an increased beneficial use of SNSs in medical education. 

## Supplementary Information

**Additional file 1: Appendix I.** The SNSME questionnaire. **Appendix II.** Pre and post workshop questionnaire. 

## Data Availability

The original data can be acquired from the corresponding author upon reasonable request through email.
