# Supplementary material for: The educational use of social networking sites among medical and health sciences students: a cross campus interventional study
Source: BMC Med Educ. 2022 Jul 3;22:525. doi: 10.1186/s12909-022-03569-3 (PMC9251038; doi:10.1186/s12909-022-03569-3)
Supplement: Supplementary file 1 — Additional file 1: Appendix I. The SNSME questionnaire. Appendix II. Pre and post workshop questionnaire. [file 12909_2022_3569_MOESM1_ESM.pdf]

Additional file 1

Appendix I. The SNSME questionnaire.

**Social Networking Sites in Medical Education (SNSME)**						
**Age (in years)**						
▪ Less than or equal to 20						
▪ 21-24						
▪ Above 25						
**Gender**						
▪ Male						
▪ Female						
**Year in MBBS course**						
1 2 3 4 5 6						
- What is your most preferred social networking site?						
**No.**	**Question**	**Never**	**Once a month**	**Once a week**	**Once a day**	**3-5 times a day**
1	How often do you use e-mail for sharing information for educational purpose?					
2	How often do you use social networking sites (e.g., Facebook, Youtube, Twitter, Linkedin, Wechat and Flickr) to keep in touch with peers and tutors?					
3	How often do you use social networking sites (i.e., Facebook, Youtube, Twitter, Linkedin, and Flickr) to share education-related information?					
4	How often do you use social networking sites (i.e., Facebook, Youtube, Twitter, Linkedin, and Flickr) for sharing research, innovations in medicine, and updates in medical field?					

5	How often do you read blogs or Wikis for education related information?					
6	How often do you contribute to blogs or Wikis to share information, or for dissemination of knowledge?					

**No.**	**Question**	**Strongly agree**	**Agree**	**Neutral**	**Disagree**	**Strongly disagree**
7	Social networking sites help me in collation of educational materials					
8	Social networking sites are helpful in collaborative and peer-to-peer learning					
9	Social networking sites are useful in developing reading and writing web skills					
10	Social networking sites provide opportunity of virtual meeting with other students and tutors					
11	Social networking sites help me to communicate with peers about class projects					
12	Social networking sites help me to access educational resources					
13	Social networking sites help me to retrieve educational references for research					
14	Social networking sites facilitate my professional					

	development of learning skills in technology					
15	Social networking sites are useful in communicating with classmates about course-related topics					
16	I have found social networking sites useful during the pre-exam period when I get an instant answer/explanation from my peer, instead of going through the books					
17	I have found social networking sites useful for sharing notes and lectures					
18	I have found social networking sites useful for educational purposes					
19	Medical students need supervision and guidance for the appropriate use of social networking sites for educational purposes					
20	I believe that social networking sites are inappropriate for sharing classroom materials, information, and discussing healthcare related topics					

Appendix II. Pre and post workshop questionnaire

Pre-Workshop Questionnaire

"Determining the impact of a guided workshop on the educational use of social networking sites among medical and health sciences students."

Choose the name of your college/university *

College of Medicine, University of Sharjah (COM-UoS)
College of Dental Medicine, University of Sharjah (CDM-UoS)
College of Health Sciences, University of Sharjah (CHS-UoS)
Ameer-ud-Din Medical College (AMC)
Universiti Sains Islam Malaysia (USIM)

Year of Study: *

Foundation Year
Year 1
Year 2
Year 3
Year 4
Year 5

Answer the following questions by choosing "Yes" or "No" *

1. Do you have a formal social media or digital health course in the existing curriculum?
2. Have you received any formal training to use Social Networking Site for education?
3. Do you have prior knowledge of Web 2.0 technology and its applications in the digital age?
4. Do you know what "digital professionalism" is?

Which of the following is your preferred social networking sites for education purpose? *

Twitter
Instagram
Facebook
Pinterest
Whatsapp
Snapchat
TikTok
Telegram

Please select your preferred educational usages of social networking sites. (You may select more than one) *

1. Class participation
2. Connection with experts in your area of study
3. Collaborative learning
4. Up-to-date information
5. Interaction among classmates/peers

6. Efficient for conceptual learning
7. Connection with community practice
8. Feedbacks
9. E-portfolio (Self-documentation of personal work and achievement)
10. Publish ideas and opinions in real-time format

Post-Workshop Questionnaire

"Determining the impact of a guided workshop on the educational use of social networking sites among medical and health sciences students."

Choose the name of your college/university *

College of Medicine, University of Sharjah (COM-UoS)
College of Dental Medicine, University of Sharjah (CDM-UoS)
College of Health Sciences, University of Sharjah (CHS-UoS)
Ameer-ud-Din Medical College (AMC)
Universiti Sains Islam Malaysia (USIM)

Year of Study: *

Foundation Year
Year 1
Year 2
Year 3
Year 4
Year 5

Answer the following questions by choosing "Yes" or "No" *

1. Do you have a formal social media or digital health course in the existing curriculum?
2. Have you received any formal training to use Social Networking Site for education?
3. Do you have prior knowledge of Web 2.0 technology and its applications in the digital age?
4. Do you know what "digital professionalism" is?
5. ***Has this workshop added to your knowledge, and understanding skills in using social networking sites for education?***

Which of the following is your preferred social networking sites for education purpose? *

Twitter
Instagram
Facebook
Pinterest
Whatsapp
Snapchat
TikTok
Telegram

Please select your preferred educational usages of social networking sites. (You may select more than one) *

1. Class participation
2. Connection with experts in your area of study
3. Collaborative learning
4. Up-to-date information
5. Interaction among classmates/peers
6. Efficient for conceptual learning
7. Connection with community practice
8. Feedbacks
9. E-portfolio (Self-documentation of personal work and achievement)
10. Publish ideas and opinions in real-time format

Please provide any remarks about today's workshop (Open ended)
